# In this month’s Bulletin

**DOI:** 10.2471/BLT.25.000125

**Published:** 2025-01-01

In the editorial section, Katherine Littler et al. (2) call for papers for a special theme issue on the ethics of research into health and climate change.

Gary Humphreys (5–6) reports on initiatives to mitigate unintended consequences of health policies. Glenda Gray talks to Humphreys (7–8) about her career working on HIV prevention and treatment.

## China

### Paediatric cancers

Lin Bai et al. (19–31) track affordability of treatment.

## Denmark, Norway, Sweden

### Older antibiotics and paediatric formulations

Christine Årdal et al. (51–56) note the implications of fragmented markets.

## South-East Asia and Western Pacific Regions

### Price controls on food

Bella Sträuli et al. (43–50) look for policy coherence in noncommunicable diseases prevention.

## Ukraine

### Functional health-care facilities

Maggie Montgomery et al. (66–71) report on fieldwork to improve water and sanitation.

## Global

### International Nonproprietary Names

Sarel F Malan et al. (9–18) trace five decades of new pharmaceutical products by disease category.

### Health financing decisions

Matthew S McCoy et al. (32–36) analyse the basis for public engagement.

### Contents of the pharmacy

Petra Brhlikova et al. (37–42) examine links between drug registers and essential medicines lists.

### Identifying factors that influence obesity prevalence

Tim Lobstein & Mojca Gabrijelčič (57–65) propose a method of policy assessment.

### Air quality standards

Yevgen Nazarenko et al. (72–73) argue for measurement of ultrafine particles.

### Predatory journals

Christina Wee at al. (74–75) list measures to prevent fraud in scientific publishing.

